# Pembrolizumab and Enfortumab-Induced Immunotherapy Myocarditis

**DOI:** 10.7759/cureus.109348

**Published:** 2026-05-21

**Authors:** Chirag Lodha, Eric J Basile, Corbin C Wright

**Affiliations:** 1 Internal Medicine, University of South Florida Morsani College of Medicine, Tampa, USA; 2 Cardiovascular Disease, University of South Florida Morsani College of Medicine, Tampa, USA

**Keywords:** enfortumab vedotin, ici myocarditis, immune-checkpoint inhibitor adverse effects, immunotherapy, myocarditis, pembrolizumab side effect

## Abstract

A 64-year-old male with coronary artery disease, hypertension, hyperlipidemia, obstructive sleep apnea, and right ureteral urothelial carcinoma presented with chest pain, neck weakness, and myalgias after his first cycle of pembrolizumab and enfortumab vedotin. He developed a non-ST-segment elevation myocardial infarction (NSTEMI) alongside suspected immune checkpoint inhibitor (ICI)-associated myocarditis and myositis. Initial tests showed elevated troponin at 568 ng/L, creatinine phosphokinase (CPK) at 3,332 U/L, and transaminases. ECG revealed anterolateral ST-segment depression and left anterior fascicular block. Cardiac catheterization confirmed 90% mid-left anterior descending (LAD) artery stenosis, treated with a drug-eluting stent, and endomyocardial biopsy verified focal moderate lymphocytic myocarditis with myocyte necrosis. Treatment with high-dose methylprednisolone and intravenous immunoglobulin led to improvement in symptoms, biomarkers, and cardiac function. He was discharged on prednisone taper, dual antiplatelet therapy, anticoagulation, and prophylaxis, with outpatient follow-up arranged.

## Introduction

Immune checkpoint inhibitors (ICI) like pembrolizumab have transformed treatment for advanced urothelial carcinoma, especially when combined with enfortumab vedotin. The EV-302/KEYNOTE-A39 trial demonstrated sustained survival benefits with this combination over chemotherapy, establishing it as first-line therapy for locally advanced or metastatic urothelial carcinoma [1]. Clinicians increasingly encounter immune-related adverse events (irAEs) with these agents, including rare but severe cardiac and muscular toxicities, such as myocarditis and myositis, as well as T-cell-mediated inflammation of other organs, such as pneumonitis. ICI-associated myocarditis emerges as a critical complication, often presenting with elevated troponins, arrhythmias, and hemodynamic instability [2]. Myositis frequently accompanies myocarditis, complicating diagnosis and management in patients with cardiovascular comorbidities [3]. This case report details non-ST-segment elevation myocardial infarction (NSTEMI), myocarditis, and myositis in a patient post-ICI initiation, highlighting diagnostic challenges and therapeutic strategies.

Literature underscores the lethality of ICI-myocarditis, with an incidence around 1% but mortality exceeding 40% in affected cases [2]. Endomyocardial biopsy remains the gold standard for confirmation, revealing lymphocytic infiltrates [4]. Combination therapies like pembrolizumab and enfortumab vedotin carry specific toxicity profiles, including skin reactions and neuropathy, though cardiac events warrant vigilance [5]. Prior cases report complete heart block or fulminant myocarditis (severe myocarditis leading to cardiogenic shock) post-pembrolizumab, emphasizing early intervention [4,6]. Patients with underlying coronary disease face amplified risks when irAEs mimic acute coronary syndromes (ACS), as they can both present with elevated troponin, ECG changes, and nonspecific chest pain or discomfort. It is therefore important to stratify the cardiovascular risk of these patients and trend troponin and symptoms to truly help determine whether they have ICI myocarditis or an ACS. This introduction frames the interplay of oncologic therapy and cardiovascular complications in this patient case.

## Case presentation

A 64-year-old male with a history of coronary artery disease, hypertension, hyperlipidemia, obstructive sleep apnea, and right ureteral urothelial carcinoma post-resection presented with chest pain, neck weakness, and myalgias after his first cycle of pembrolizumab and enfortumab vedotin. His vitals were significant for a blood pressure of 135/85, a heart rate of 78 beats per minute, and an oxygen saturation of 98% on room air. Admission labs revealed troponin of 568 ng/L, which downtrended to within normal limits six hours afterward, creatinine phosphokinase (CPK) of 3,332 U/L, and elevated transaminases (Table 1). ECG showed anterolateral ST depression and left anterior fascicular block (Figure 1).

**Table 1:** Patient laboratory values on presentation CPK: creatinine phosphokinase

Lab Values	Patient Value	Normal Value
Troponin (ng/L)	568	<50
CPK (U/L)	3,332	<30

**Figure 1:**
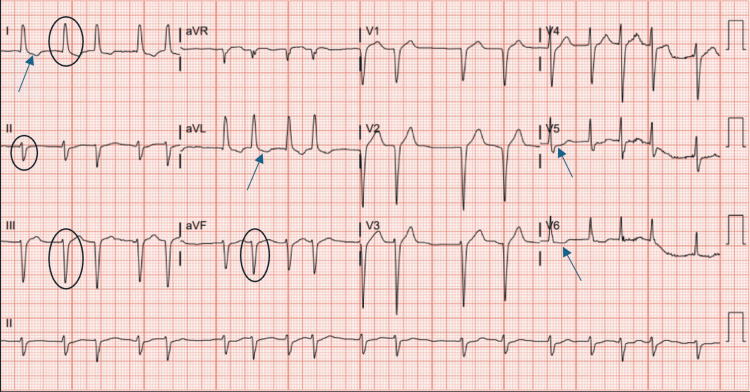
ECG showed anterolateral ST depression and left anterior fascicular block Arrows showing ST-depression in leads I, aVL, V5, V6. Circles indicating left anterior fascicular block.

His physical exam was significant for 4/5 muscle strength in the bilateral lower extremities. Cardiac auscultation had no murmurs or rubs, and lungs were clear to auscultation bilaterally. Transthoracic echocardiogram demonstrated normal left ventricular ejection fraction (LVEF) (EF 55-60%) and mild aortic stenosis. A CT angiogram ruled out pulmonary embolism or intrathoracic pathology. The presentation raised concerns for NSTEMI amid suspected ICI toxicity, including myocarditis, myositis, and possible myasthenia gravis. One day later, he underwent left and right heart catheterization, revealing 90% mid-left anterior descending (LAD) artery stenosis. Interventional cardiology placed a drug-eluting stent in the LAD and obtained endomyocardial biopsy samples. Biopsy confirmed focal moderate lymphocytic myocarditis with myocyte necrosis, absent giant cells, granulomas, or viral inclusions, consistent with ICI association (Figure 2). 

**Figure 2:**
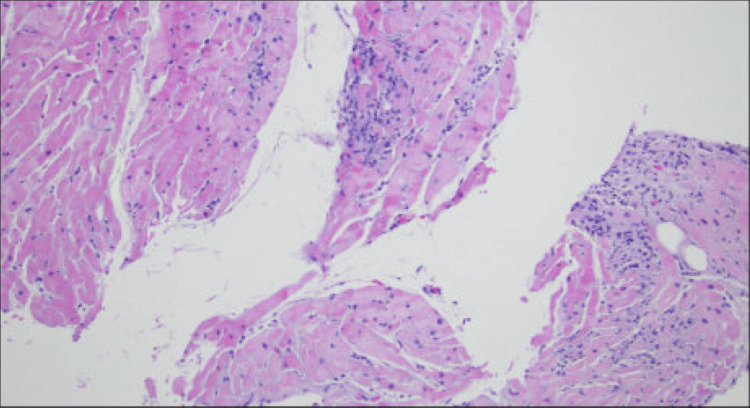
Pathology showing focal moderate lymphocytic myocarditis with intact and scarred interstitium with myocyte necrosis

During the procedure, new-onset atrial fibrillation with rapid ventricular response occurred; amiodarone and metoprolol achieved cardioversion to sinus rhythm. He continued dual antiplatelet therapy and transitioned from heparin to apixaban post-acutely. Cardiac MRI later showed LVEF 46% with hypokinesis of the mid-anterior septum and distal septal wall, without inflammation or infarct.

Neurology evaluated for myasthenia gravis, but serial exams and serologies proved negative. Treatment followed oncology and neuro-oncology guidelines with high-dose intravenous methylprednisolone (1 g daily, then 500 mg every other day for 5 g total) alternated with IVIG. He received five intravenous immunoglobulin (IVIG) doses and six methylprednisolone doses, planning a prednisone taper thereafter. His CPK and troponin decreased to normal limits within two days of methylprednisolone treatment, with chest pain and weakness improving simultaneously, although mild neck weakness persisted. He remained hemodynamically stable in sinus rhythm and ambulated independently. Secondary issues included managed coronary disease, held atorvastatin for transaminitis, and new right hydroureteronephrosis. 

## Discussion

This case illustrates the diagnostic and therapeutic complexities of ICI-associated myocarditis complicating NSTEMI in a patient on pembrolizumab and enfortumab vedotin for urothelial carcinoma [1]. Elevated troponins and CPK, alongside biopsy-proven lymphocytic infiltrates, aligned with established histopathological features of ICI-myocarditis [2]. The temporal link to first-cycle therapy, with symptoms of chest pain and myalgias, mirrored reports of early-onset irAEs [4]. Pre-existing coronary disease obscured differentiation between ischemic and immune-mediated injury, necessitating catheterization and biopsy for clarity. Although there was a high-grade stenotic lesion, it was stable coronary disease, and the timeframe of his symptoms did align with the timeframe of the ICI treatment infusions. Additionally, the chest pain resolved after two days of treatment, rather than acutely after receiving a stent to the LAD, further supporting the diagnosis of ICI myocarditis. This, in conjunction with myalgias and vision difficulties, ultimately led to the diagnosis of ICI myocarditis. Cardiac MRI has a high sensitivity for detecting myocarditis, although endomyocardial biopsy is the gold standard. MRI is also not as sensitive in the early stages of inflammation, as imaging tends to lag behind clinical presentation [3,4]. The patient's findings of reduced EF and regional hypokinesis further supported myocarditis, despite lacking overt inflammation [2]. Multidisciplinary input proved essential in navigating these overlapping pathologies. 

Management with high-dose steroids and IVIG yielded biomarker normalization and symptom resolution, consistent with guidelines for grades 3-4 ICI-myocarditis [2,7]. Atrial fibrillation during catheterization required prompt rhythm control, avoiding escalation seen in fulminant cases needing ECMO [8]. Negative myasthenia gravis workup ruled out neuromuscular mimicry, focusing therapy on myocarditis and myositis. Literature highlights steroid-refractory cases benefiting from ruxolitinib or abatacept, though our patient responded to initial immunosuppression [7,9]. Enfortumab vedotin-pembrolizumab combinations carry cutaneous toxicities, but cardiac events remain rarer, underscoring vigilance needed with these therapies [5,10]. Prior fatal myocarditis post-pembrolizumab cases warn of potential severity [6,11]. Our biopsy excluded infectious or giant cell variants, affirming immune etiology. 

Patient comorbidities amplified risks; coronary stenosis demanded stenting amid anticoagulation challenges [12]. Transaminitis prompted statin hold, balancing lipid control with hepatotoxicity. Prophylaxis for *Pneumocystis jiroveciipneumonia* addressed immunosuppression risks. Outcomes improved with therapy, contrasting fatal cases without biopsy confirmation [6]. This report reinforces early biopsy, high-dose steroids, and IVIG as cornerstones, with close monitoring for arrhythmias [4]. Future research should clarify combination therapy risks and optimal second-line agents, as well as further delineation between ACS and myocarditis upon presentation [7,9]. A key limitation of this case report is that it is one isolated case, and therefore, its conclusions cannot be generalized to a larger patient population. Larger-scale trials and retrospective analyses must be done in order to further investigate optimal therapies and management.

## Conclusions

This case underscores the need for rapid recognition of ICI-associated myocarditis and myositis in patients receiving pembrolizumab and enfortumab vedotin. Multidisciplinary collaboration facilitated precise diagnosis via biopsy and effective treatment with steroids and IVIG. The patient's recovery, including biomarker normalization and functional improvement, highlights the potential for favorable outcomes with prompt intervention. Discharge planning with taper, rehabilitation, and follow-up ensured sustained stability. Clinicians must maintain high suspicion for irAEs in similar presentations, particularly with cardiovascular history. Continued reporting of such cases will refine management strategies for these evolving toxicities. 

## References

[1] Powles T, Valderrama BP, Gupta S (2024). Enfortumab vedotin and pembrolizumab in untreated advanced urothelial cancer. N Engl J Med.

[2] Palaskas N, Lopez-Mattei J, Durand JB, Iliescu C, Deswal A (2020). Immune checkpoint inhibitor myocarditis: pathophysiological characteristics, diagnosis, and treatment. J Am Heart Assoc.

[3] Sánchez-Camacho A, Torres-Zurita A, Gallego-López L (2025). Management of immune-related myocarditis, myositis and myasthenia gravis (MMM) overlap syndrome: a single institution case series and literature review. Front Immunol.

[4] Panchal V, Momin E, Jain S, Singh A, Ghuman G, Brar V (2025). Immune checkpoint inhibitor-induced myocarditis: a case report of complete heart block and challenges in a patient on pembrolizumab. J Innov Card Rhythm Manag.

[5] Simões CA, Asbeg N, Santos ACFdF, Cavalcante D, de Jesus ACVdS, Gazzoni G, Stecca C (2025). Toxicity profile of enfortumab vedotin with or without pembrolizumab in patients with metastatic urothelial carcinoma: a systematic review and meta-analysis. J Clin Oncol.

[6] Cavalcante L, Chandana S, Lakhani N (2024). Case report of fatal immune-mediated myocarditis following treatment with davoceticept (ALPN-202), a PD-L1-dependent CD28 costimulator and dual PD-L1/CTLA-4 checkpoint inhibitor, in combination with pembrolizumab. J Immunother Cancer.

[7] Wadden E, Lai C, Grivas P (2025). Successful treatment of immune checkpoint inhibitor-associated fulminant myocarditis with abatacept and ruxolitinib: a case report. Eur Heart J Case Rep.

[8] Zhang Y, Li R, Jiang Y, Sun S, Yuan Y, Shang Y, Shu H (2025). Successful ECMO support for cardiogenic shock induced by immune checkpoint inhibitor-associated myocarditis: a case report and literature review. Front Immunol.

[9] Vockenhuber T, Baldinger L, Clausen J (2025). Successful use of ruxolitinib for steroid-refractory immune checkpoint inhibitor-associated myocarditis. JACC Case Rep.

[10] Müller B, Curatolo R, Juratli HA, Husic A, Nehring J, Potlukova E, Kohler A (2024). Severe cutaneous toxicity in a 67-year-old patient with metastatic urothelial carcinoma undergoing therapy with enfortumab vedotin and pembrolizumab. Eur J Case Rep Intern Med.

[11] Hashimoto T, Takahashi K, Ito K (2024). An autopsy case of lung adenocarcinoma with immune checkpoint inhibitor-induced pneumonia and fulminant myocarditis following pembrolizumab administration: a case report. Int Cancer Conf J.

[12] Mitrani L, Jou S, Mendez S, Song C, Yoon J, Dangayach NS, Sahni G (2023). Abstract 16013: non-bacterial thrombotic endocarditis and recurrent cerebrovascular accidents in immune checkpoint inhibitor myocarditis. Circulation.

